# Understanding the spread of infectious diseases in edge areas of hotspots: dengue epidemics in tropical metropolitan regions

**DOI:** 10.1186/s12942-023-00355-2

**Published:** 2023-12-10

**Authors:** Ya-Peng Lee, Tzai-Hung Wen

**Affiliations:** 1 Department of Geography, National Taiwan University, Taipei, Taiwan; 2 National Science and Technology Center for Disaster Reduction, Taipei, Taiwan

**Keywords:** Spatial epidemiology, Conditional autoregressive model, Bayesian areal Wombling methods, Spatial panel model, Disease mapping

## Abstract

Identifying clusters or hotspots from disease maps is critical in research and practice. Hotspots have been shown to have a higher potential for transmission risk and may be the source of infections, making them a priority for controlling epidemics. However, the role of edge areas of hotspots in disease transmission remains unclear. This study aims to investigate the role of edge areas in disease transmission by examining whether disease incidence rate growth is higher in the edges of disease hotspots during outbreaks. Our data is based on the three most severe dengue epidemic years in Kaohsiung city, Taiwan, from 1998 to 2020. We employed conditional autoregressive (CAR) models and Bayesian areal Wombling methods to identify significant edge areas of hotspots based on the extent of risk difference between adjacent areas. The difference-in-difference (DID) estimator in spatial panel models measures the growth rate of risk by comparing the incidence rate between two groups (hotspots and edge areas) over two time periods. Our results show that in years characterized by exceptionally large-scale outbreaks, the edge areas of hotspots have a more significant increase in disease risk than hotspots, leading to a higher risk of disease transmission and potential disease foci. This finding explains the geographic diffusion mechanism of epidemics, a pattern mixed with expansion and relocation, indicating that the edge areas play an essential role. The study highlights the importance of considering edge areas of hotspots in disease transmission. Furthermore, it provides valuable insights for policymakers and health authorities in designing effective interventions to control large-scale disease outbreaks.

## Introduction

Disease mapping, an essential approach for understanding and addressing infectious diseases, examines changes in the risk or occurrence of epidemics in geographic space and their temporal evolution. Visualizing complex disease information and using spatial smoothing methods to reveal trends and patterns of disease incidence enables the delimitation of boundaries around high-incidence areas for disease prevention and control. Identifying clusters or hotspots from disease maps is crucial to research and practice. It allows health authorities to understand high-risk regions and policymakers to implement targeted interventions. Spatial statistical methods for hotspot exploration and detection, such as kernel density estimation, Moran's I and Gi* approaches, can be used for identifying the areas where the number of infected cases is concentrated in a few locations and exceeds expected values [4, 9]. Recent studies have shown that hotspots have a higher potential for transmission risk and may be the source of infections, making them a priority for controlling epidemics [20, 27].

However, there is a lack of research on risk assessment in the edge areas surrounding disease hotspots, which may have a higher proportion of interactions between susceptible hosts and infective cases. These areas may be vulnerable to infection without movement restriction, leading to increased infections and disease spread due to high-frequent contact. Some studies have compared the risk patterns in the clusters and their surrounding areas, but more research is needed to confirm these findings through quantitative models. For example, in the western highlands of Kenya, interventions in malaria hotspots reduced prevalence only within the hotspot itself, with no decline in surrounding areas after eight weeks and no sustained decline in the hotspot after 16 weeks [6]. Similarly, in southern Taiwan, superspreaders of dengue moved from the center of hotspots to the border and then outside over time during an exponential growth stage [26]. These studies provide descriptive comparisons, but further investigation is needed to confirm these findings and understand the role of edge areas in disease transmission.

Therefore, this study aims to establish a framework for examining whether disease incidence rate growth would be higher in edge areas during outbreaks by delimiting the disease hotspot and its edge areas. Using conditional autoregressive (CAR) models, we divide the study area into hotspot areas and their edge areas and Bayesian areal Wombling methods to identify significant edge areas based on the extent of risk difference between adjacent hotspots. Then, we compare the growth of disease risk in edge areas using difference in difference (DID) estimation from a spatial panel model. Our study will provide a better understanding of the role of edge areas in the spread of infectious diseases and inform the spatial targeting of interventions in outbreaks. The case study used is the large-scale outbreaks of dengue fever epidemics from 1990–2020 in a tropical metropolitan area, Kaohsiung, Taiwan.

## Data and methods

### Study area

Kaohsiung City, located in tropical regions, is Taiwan's third most populous metropolitan area. The tropical monsoon climate, characterized by high humidity and temperature, creates ideal conditions for transmitting the dengue virus [21, 28]. With an average monthly temperature of 25.4 °C and concentrated rainfall of 1968.2 mm from May to September [31], the area is conducive to the breeding of vector mosquitoes, *Aedes aegypti* and *Aedes albopictus*, leading to frequent dengue outbreaks in the past 20 years [38].

**Fig. 1:**
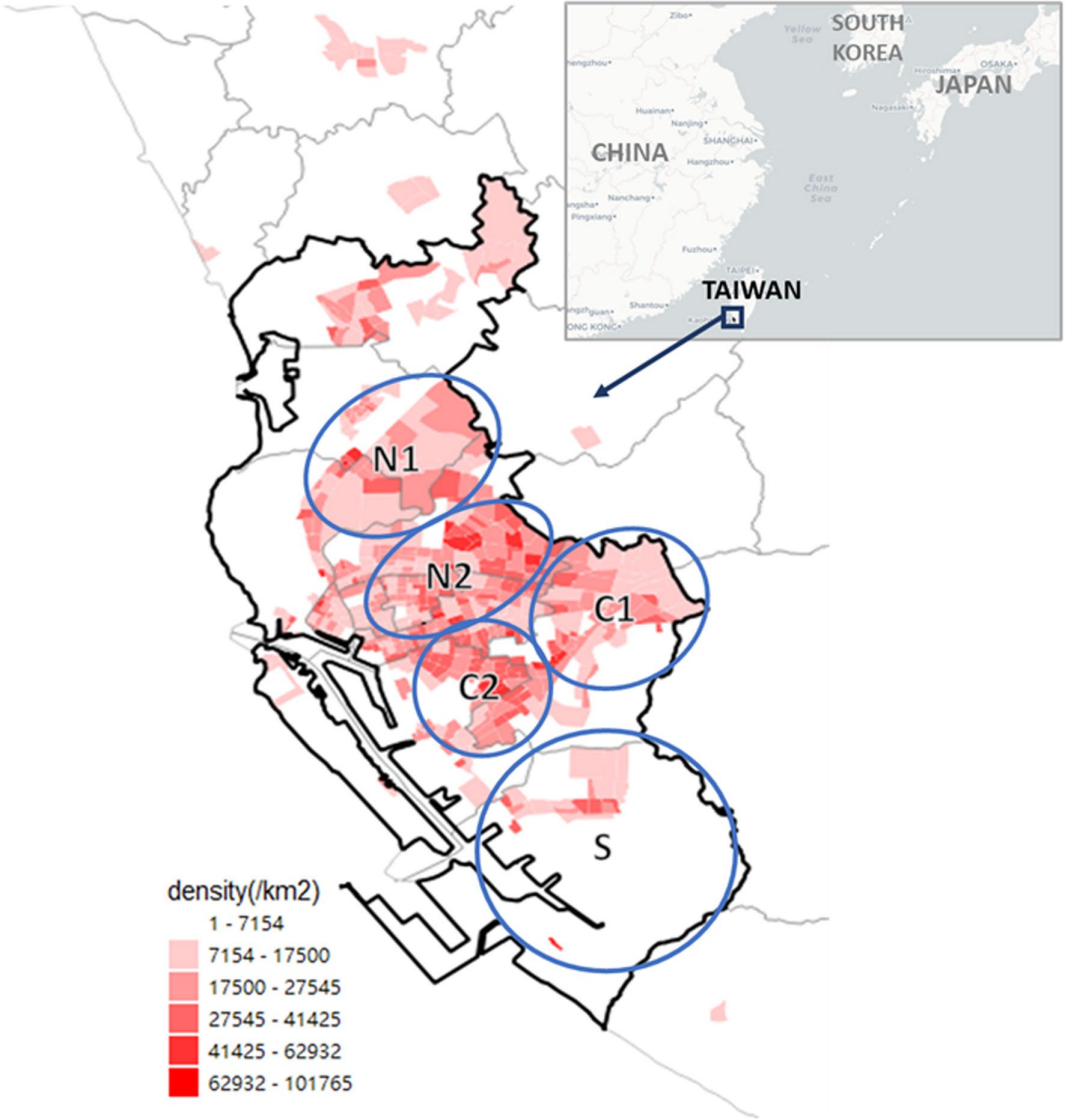
Study area. The subgraph on the top-right shows the location of the study area relative to other countries in East Asia. And the main graph shows the main area in Kaohsiung city, Taiwan and the population density in each village. According to the concentration of population density distribution and geographical location, we have hightlighted the five regions (N1, N2, C1, C2, and S) in blue circles shown on the graph in the study area to discuss the hotspot and edge area distribution

The study area is focused on the urban core of Kaohsiung, including the Fongshan district and other major metropolitan areas (Fig. 1). The population density in this area is 9097 people/${\mathrm{km}}^{2}$, with an average of 20,174 people/${\mathrm{km}}^{2}$ and a standard deviation of 12,398 people/${\mathrm{km}}^{2}$ across villages. The village serves as the primary unit of analysis, with 528 villages in 12 districts, spanning a total area of 205 ${\mathrm{km}}^{2}$, and an average size of 0.4 ${\mathrm{km}}^{2}$ per village. To facilitate visualization and comparisons of hotspot and edge area distributions in the study area, we have highlighted the five regions (Region N1, N2, C1, C2, and S) using blue circles in Fig. 1. These regions were selected based on considerations of population density and the home range of residents within the city.

### Disease data

This study utilizes daily confirmed dengue data obtained from the Taiwan Centers for Disease Control (Taiwan-CDC), covering a period from 1998–2020. This dataset enables the examination of different outbreak scenarios at varying scales. The data fields for each case include the dates of illness onset and notification, the age of the infected individual, the village of residence, and whether the case is indigenous or imported.

Each confirmed dengue case from Taiwan-CDC is tested through laboratory diagnosis. A confirmed case is defined as: (i) positive for dengue virus isolation; or (ii) positive for dengue virus genomic sequence; or (iii) positive for non-structural protein 1 (NS1) by serum antigen test (iv) four-fold increase of dengue virus-specific IgM or IgG antibody in paired serum samples [30].

**Fig. 2:**
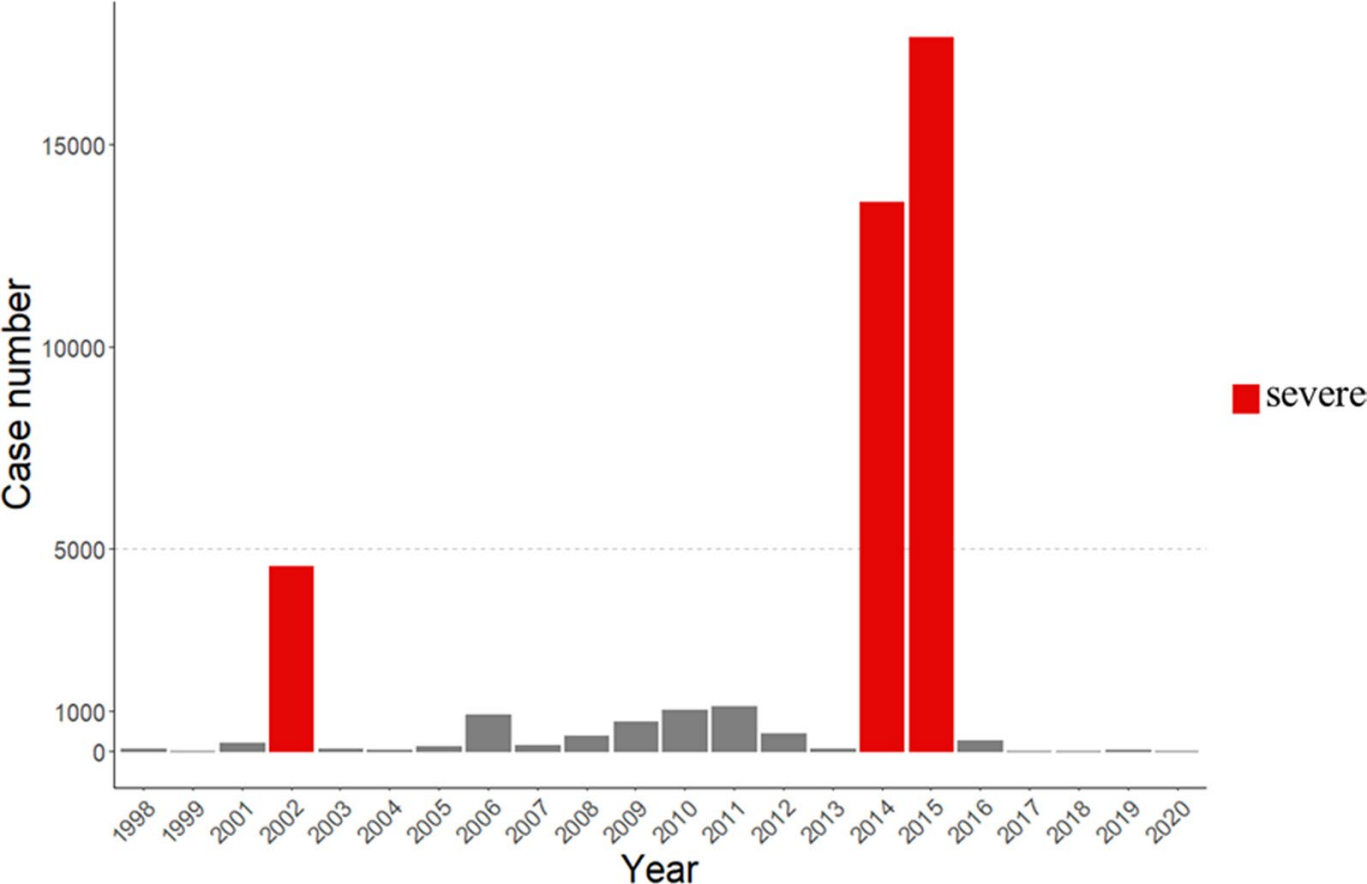
Yearly dengue indigenous case number in the study area

**Fig. 3:**
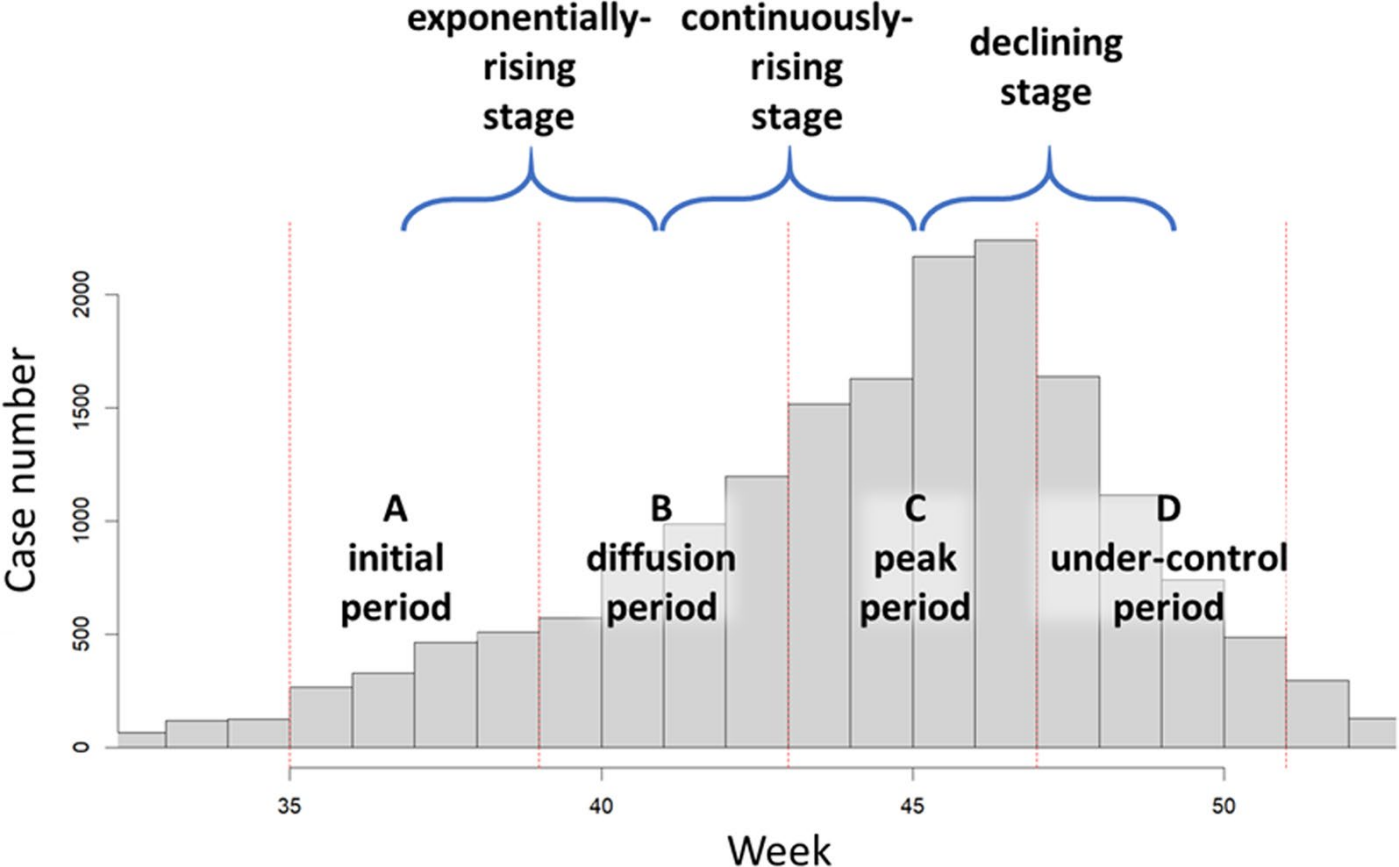
An example of dividing weekly data into periods

In this study, we focus on the dengue cases from severe dengue epidemics to examine spatial variations in disease risk. Using data from 1998 to 2020, we identified three particularly severe years: 2002, 2014, and 2015 (represented by red bars in Fig. 2). To examine temporal variations within each epidemic, we divided each year into four periods: the initial period (A), diffusion period (B), peak period (C), and under-control period (D) according to the growth curve of large-scale outbreaks. Each period lasted four weeks, and the number of indigenous cases in each period were aggregated to determine hotspot and boundary detection. The selection of a four-week time period is based on the average generation time of dengue epidemics, typically spans from 16 to 34 days [15], allowing us to capture the disease dynamics and progression effectively. We also defined the epidemic stages as the transition between consecutive periods, including the exponentially-rising stage (A to B), continuously-rising stage (B to C), and declining stage (C to D) (as shown in Fig. 3).

### Methods

#### Study framework

**Fig. 4:**
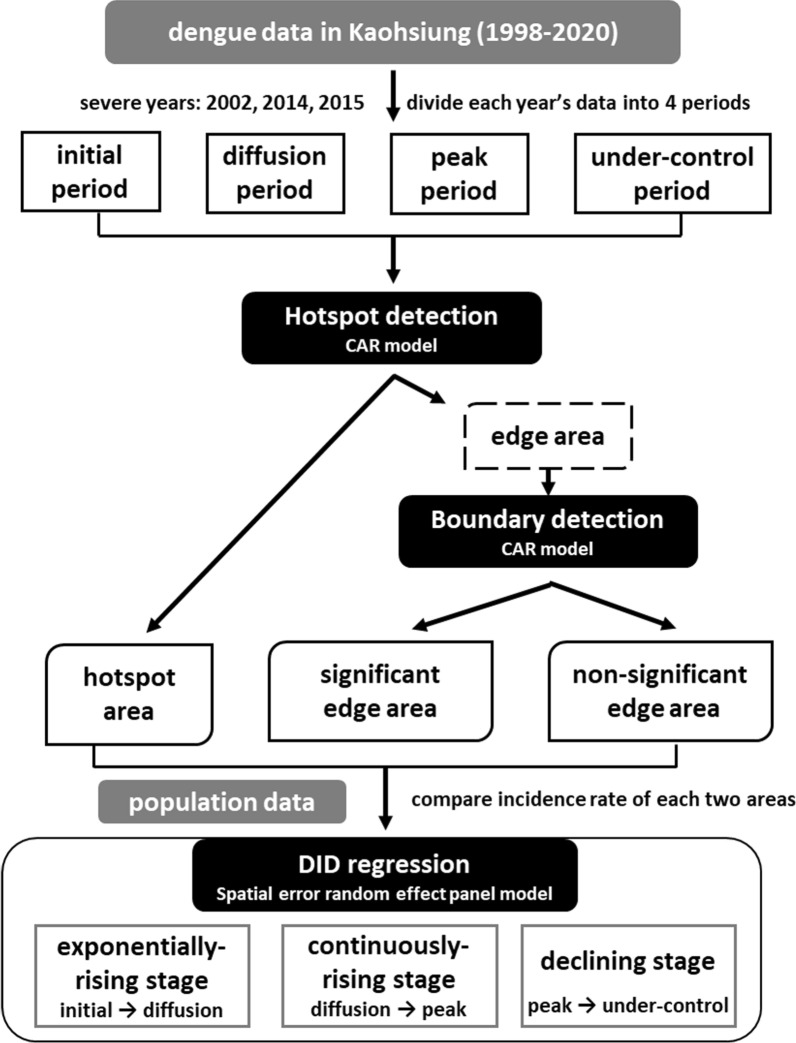
Research framework

The research framework, illustrated in Fig. 4, consists of three key steps. First, the weekly epidemic data from the selected years is divided into four periods. Next, the hotspots and edge areas are detected using a conditional autoregressive (CAR) model. Finally, we assess whether the risk of dengue transmission is higher at the edge areas of a cluster compared to the cluster itself during different stages of each selected year. The difference-in-difference (DID) effect is estimated by comparing the incidence rates between hotspots and edge areas with a spatial error random effect panel model.

#### Statistical methods

##### CAR model for hotspot detection

The hotspots are detected using the CAR model [19]. The model utilizes the number of indigenous confirmed cases of dengue in each village, represented as ${Y}_{k}$, to estimate the probability of zero-based point mass distribution (${\omega }_{k}$) and mean of the mixed distribution (${\mu }_{k}$). The data likelihood model used is a zero-inflated Poisson model, which combines the excess of zero infections in many villages and the count data of dengue cases. It is assumed to be a joint point mass distribution based on the logistic distribution and a Poisson distribution (Eq. 1).

1 $${Y}_{k} \sim \mathrm{ ZIP}({\mu }_{k},{\omega }_{k})$$

Then, a linear equation, Eq. 2, is used to estimate the number of cases in each village. ${O}_{k}$ is the intercept, $\beta$ is the regression coefficient, and ${x}_{k}$ is the population in $k$th village because there would be more cases in a place with more people.${\psi }_{k}$ is given as CAR prior, which stands for a spatial random effect. The CAR prior (Eq. 3) includes only a single set of random effects ${\varphi }_{k}$. It is given the condition that corresponds to a normal distribution, and a village *k* is affected by all the neighbors except itself (${\varphi }_{-k}$). Here, movement of daily life within short distances is assumed to build the adjacent matrix *W*. The definition of "neighbor" relies on Rook contiguity, which is underpinned by the assumption that individuals typically move within their neighboring villages in their daily routines. If two villages share the same edge, then ${w}_{ki}=1$; otherwise, if two villages are not adjacent, then ${w}_{ki}=0$. Also, there are two parameters $\rho$ and ${\tau }^{2}$ to be estimated. $\rho$ represents the parameter of spatial dependence. If $\rho$=0, the value between each village is independent; if $\rho$=1, there is spatial autocorrelation, and it corresponds to the intrinsic CAR model. ${\tau }^{2}$ is proportional to the concentration of this normal distribution.

2 $$\mathrm{ln}\left({\mu }_{k}\right)= {x}_{k}\beta + {O}_{k}+ {\psi }_{k}$$

3 $${\psi }_{k}= {\varphi }_{k}$$

$${\varphi }_{k}| {\varphi }_{-k}, W, {\tau }^{2}, \rho \sim N(\frac{\rho {\sum }_{i=1}^{K}{w}_{ki}{\varphi }_{i}}{\rho {\sum }_{i=1}^{K}{w}_{ki} + 1 - \rho }, \frac{ {\tau }^{2}}{\rho {\sum }_{i=1}^{K}{w}_{ki} + 1 - \rho })$$

$${\tau }^{2}\sim \mathrm{Inverse}-\mathrm{Gamma }(1, 0.01)$$

$$\rho \sim \mathrm{Uniform }(0, 1)$$

Hotspot probabilities (${\widehat{p}}_{i}$) are estimated straightly by ${{\mu }_{i}}^{(g)}$, meaning the probabilities, which are estimated cases in each village, are greater than ${c}_{1}$ (Eq. 4). And ${c}_{1}$ twice the average cases in each village is used as the threshold [35] in the calculated period. From the posterior distribution p(${\mu }_{i}\left|\mathrm{y}\right)$, there would be G samples ${{\mu }_{i}}^{(g)}$ (g = 1…G), indicating the predicted case number of each village *i* after computing the CAR model by a Monte Carlo Makov Chain (MCMC). In this study, G is set up to be 100, so each village has 100 estimated case numbers. Next, a village with a hotspot probability greater than or equal to *P* (*P*≧0.7) is defined as a hotspot area, and its neighbors are called the edge areas.

4 $${\widehat{p}}_{i}\equiv \widehat{P}\left({\mu }_{i}>{c}_{1}|y\right)= \frac{{{\mu }_{i}}^{(g)}>{c}_{1}}{G}$$

##### Bayesian areal Wombling for boundary detection

The boundary detection method was first introduced by Womble [37] to measure the rate of species movement. Later, it was expanded upon by Barbujani et al. [2] and Bocquet-Appel and Bacro [5] for point or grid data analysis. For taking spatial autocorrelation and uncertainty into account, a Bayesian areal Wombling approach was developed using an intrinsic conditional autoregressive (CAR) prior as a spatial random effect [7, 22, 23, 33].

Bayesian areal Wombling is used to detect the boundary on the edges of the hotspot areas, also based on the CAR model as mentioned previously. From the posterior distribution of the CAR model, the absolute difference value between each village of the hotspot area and its each edge area neighbor would be the boundary likelihood value (${\Delta }_{ij}$), which is the case difference between two neighboring villages between a hotspot area and an edge area (Eq. 5).

5 $${{\Delta }_{ij}}^{(g)}=\left|{{\mu }_{i}}^{\left(g\right)}-{{\mu }_{j}}^{\left(g\right)}\right|$$

6 $${\widehat{p}}_{ij}\equiv \widehat{P}\left({\Delta }_{ij}>{c}_{2}|y\right)= \frac{{{\Delta }_{ij}}^{(g)}>{c}_{2}}{G}$$

**Fig. 5:**
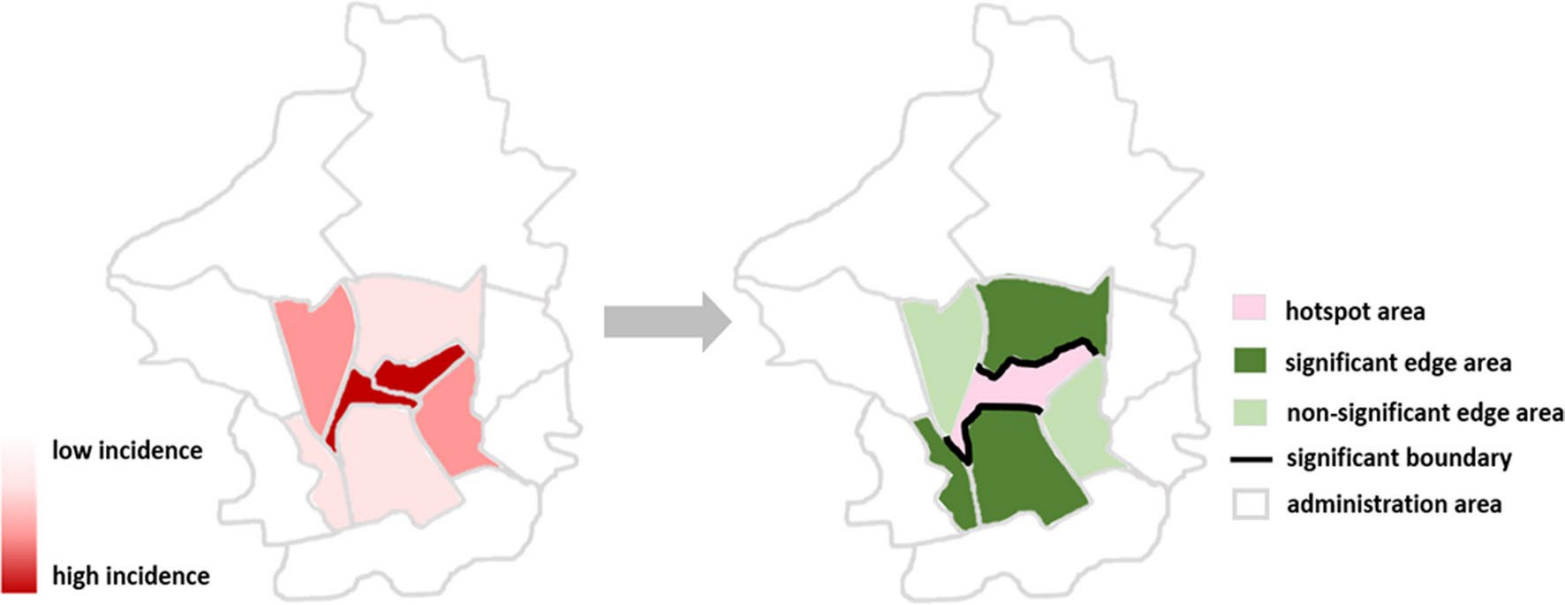
Definition of the 3 areas. The left is a schematic graph of incidence choropleth map, the darker the red it, the more confirmed cases, and vice versa. The right is a schematic graph for delimiting hotspot area, significant edge area, and non-significant edge area

Boundary probabilities (${\widehat{p}}_{ij}$) are estimated by counting the number of ${{\Delta }_{ij}}^{(g)}$ in G samples that exceed a threshold ${c}_{2}$, meaning that the difference between each pair of neighbors is more than ${c}_{2}$ cases (Eq. 6). For boundary detection, twice the average cases in each village is also used as the threshold in the boundary probability. Each edge between a hotspot area and an edge area would obtain a boundary probability. Then, an edge area village that shares a boundary probability greater than or equal to *P* (*P*≧0.7) with a hotspot is defined as a significant edge area (Fig. 5). The remaining edge areas would be defined as non-significant edge areas if they are not categorized as significant edge areas. The distinction between significant edge areas and non-significant edge areas lies in their proximity to hotspots. Significant edge areas exhibit a statistically significant disease boundary with neighboring hotspots, resulting in a notable difference in confirmed cases. This aims to observe whether there is a difference between each edge area adjacent to hotspots, and two types of edge areas.

##### Difference-in-difference estimation (DID)

The difference-in-difference (DID) method is a commonly used technique in quasi-experimental research to assess the impact of specific changes, such as a new policies or medical treatments, across multiple groups and time periods [1, 36]. This design involves two groups (control and treatment) and two time periods, assuming that the trends of the groups would be similar without any intervention or treatment [10, 36]. In our study, we make the assumption that the trends in different areas are the same. The underlying rationale behind the "same trend" assumption is to designate one area, particularly the hotspot area, as a reference point for comparison. This approach enables us to assess whether other areas exhibit a trend of increasing rates exceeding that of the hotspot area, thereby identifying a significant DID effect.

**Fig. 6:**
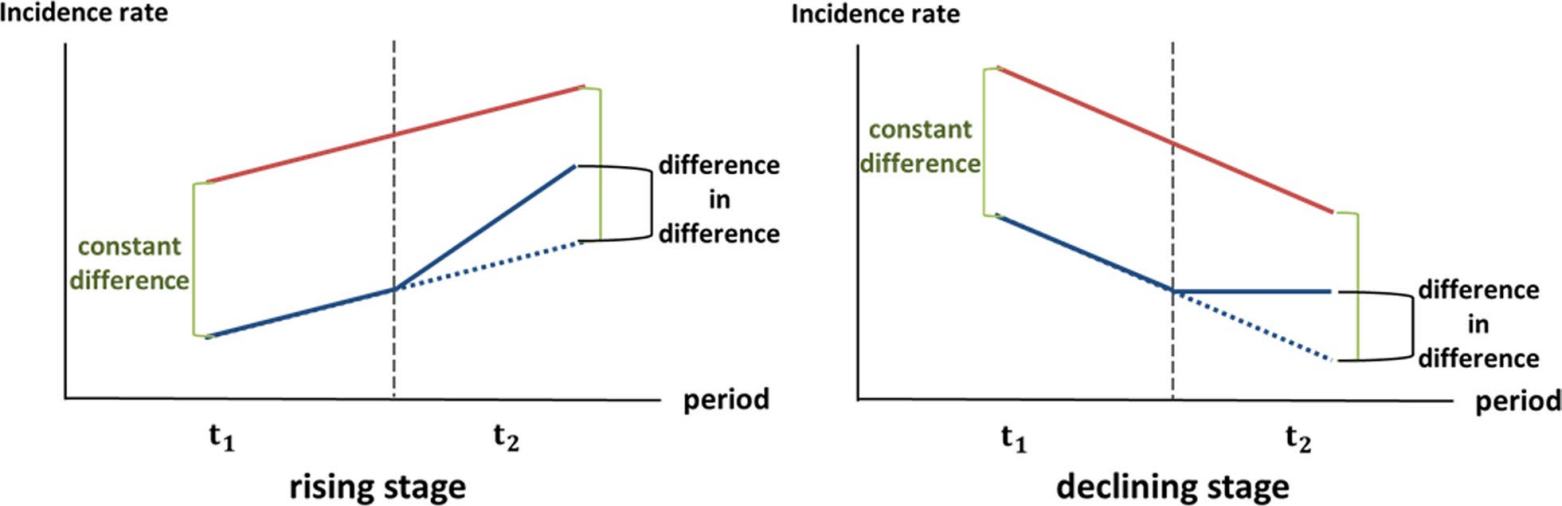
Concept of difference-in-difference

DID analysis involve two differences: the difference in time or the natural trend of the incidence rate from one period (${\mathrm{t}}_{1}$) to another (${\mathrm{t}}_{2}$) (as shown by the difference between the black dotted lines in Fig. 6), and the difference in the incidence rate between the two areas (constant difference in Fig. 6). The final result of the DID analysis is the difference between the original trend in the period ${\mathrm{t}}_{2}$ and the specific area (difference in difference in Fig. 6). Figure 6 demonstrates the situation in which the disease trend is either increasing or decreasing, with a positive value of the DID estimator.

Following the identification of hotspot areas, significant edge areas, and non-significant edge areas in each period, we applied DID regression to compare the two area groups. It is to determine if the incidence rate in the edge area is significantly higher than in the hotspot area and to compare differences between the two types of edge areas. The DID regression utilizes the spatial error random effect panel model, as described in the following equations (Eqs. 7–9).

7 $$y={\beta }_{0}+({\upiota }_{\mathrm{T}}\otimes GROUP){\beta }_{1}+(TIME\otimes GROUP){\beta }_{2}+u$$

8 $$u= \rho ({\mathrm{I}}_{\mathrm{T}}\otimes {W}_{N})u+\varepsilon$$

9 $$\varepsilon =({\upiota }_{\mathrm{T}}\otimes {\mathrm{I}}_{N})\mu +v$$

The spatial error random effect panel model proposed by Kapoor et al. [17] considers potential spatial autocorrelation in spatial data. The dependent variable, *y*, is a vector of incidence rates in each village at two time periods (Eq. 7). *GROUP* is a dummy variable comparing two of the three area groups, and *TIME* is a dummy variable indicating the pre- or post-period. The coefficients ${\beta }_{0}$, ${\beta }_{1}$, and ${\beta }_{2}$ represent the intercept, the total difference between area groups, and difference-in-difference between time and area. ${\mathrm{I}}_{\mathrm{T}}$ is an identity matrix of dimension *T*, and ${W}_{N}$ is a spatial weights matrix in an N × N form built by Rook contiguity. The disturbance vector, *u*, contains two terms—a spatial autoregressive parameter (ρ) ranging from -1 to 1, and cross-sectional specific effects ($\mu$). ${\upiota }_{\mathrm{T}}$ is a* T* × 1 vector of ones, and ${\mathrm{I}}_{N}$ is a N × N identity matrix, and $v$ is the innovation varying over different villages and periods. ${\varepsilon }_{it}$, ${\mu }_{it}$ and ${v}_{it}$ should all be independent and identically distributed (i.i.d). The main result of concern is the significance of ${\beta }_{2}$ (DID estimator), which indicates if the change in incidence rate in one area is greater than another.

The DID estimator (coefficient ${\beta }_{2}$) measures the growth rate of risk by comparing the incidence rate between two groups (hotspot and significant edge area) over two time periods (${\mathrm{t}}_{1}$ and ${\mathrm{t}}_{2}$). The model determines if the change in incidence rate in one area is statistically greater than in another. The comparison is made between the incidence rates in the same areas in both periods, with the significant edge area represented as 1 (*GROUP* = 1) and the hotspot area as 0 (*GROUP* = 0). The main result of interest is the significance of the DID estimator.

All statistical analyses were performed in R version 4.2.1. The “CARBayes” package was used for CAR models and Bayesian areal Wombling [18], and the “splm” package was used for DID estimation [25].

#### Sensitivity analysis of parameter settings

We conducted sensitivity tests for identifying hotspots and boundaries with different thresholds and probabilities. First, the values for the boundary and hotspot detection thresholds (*c*) were set in the range of 1.0 to 3.0, with a fixed probability of boundary (${\widehat{p}}_{ij}$) and hotspot (${\widehat{p}}_{i}$) of 0.7 (*P* = 0.7). These thresholds correspond to a multiple (*c* times) of the average cases in the study area during a specific period. Second, we varied *P* from 0.5 to 0.9 with fixed thresholds (*c*) at 2.0 to determine the robustness of results under different parameter settings.

## Results

### Pattern description

**Fig. 7:**
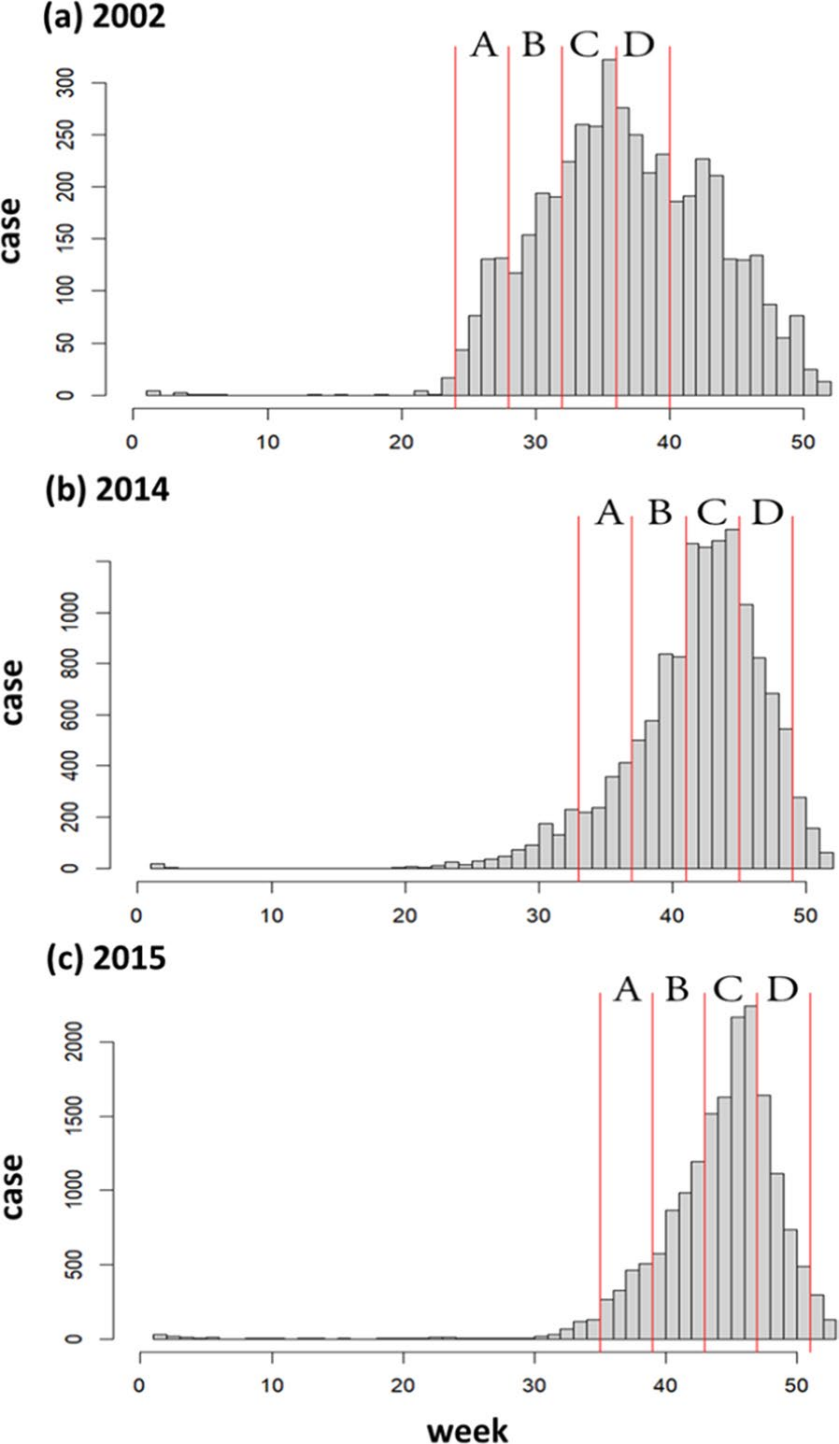
Histogram of dengue case by week in each selected year. The red lines in each subgraph indicate how periods are cut, and the A, B, C, D stands for initial, diffusion, peak, and under-control periods in order

The weekly dengue case numbers in each selected year are shown in Fig. 7, divided into four periods: initial (A), diffusion (B), peak (C), and under-control (D). Over the past three severe years, the start of the epidemic was between the 20th and 30th weeks of the year, between mid-May and the end of July. The peak season occurred between the 40th and 45th week, corresponding to October to November. However, in 2002, the pattern was slightly different from 2014 and 2015, as the case numbers were lower and did not show an exponential decline in the under-control period (D), instead continuing to decrease gradually until the end of the year. Nevertheless, the overall upward trend in weekly cases is in line with the exponential growth seen in the severe years (Fig. 7a–c). While the period cutting standard for each selected year may not perfectly align with its period name, having a uniform standard is necessary for comparisons in further discussion.

**Fig. 8:**
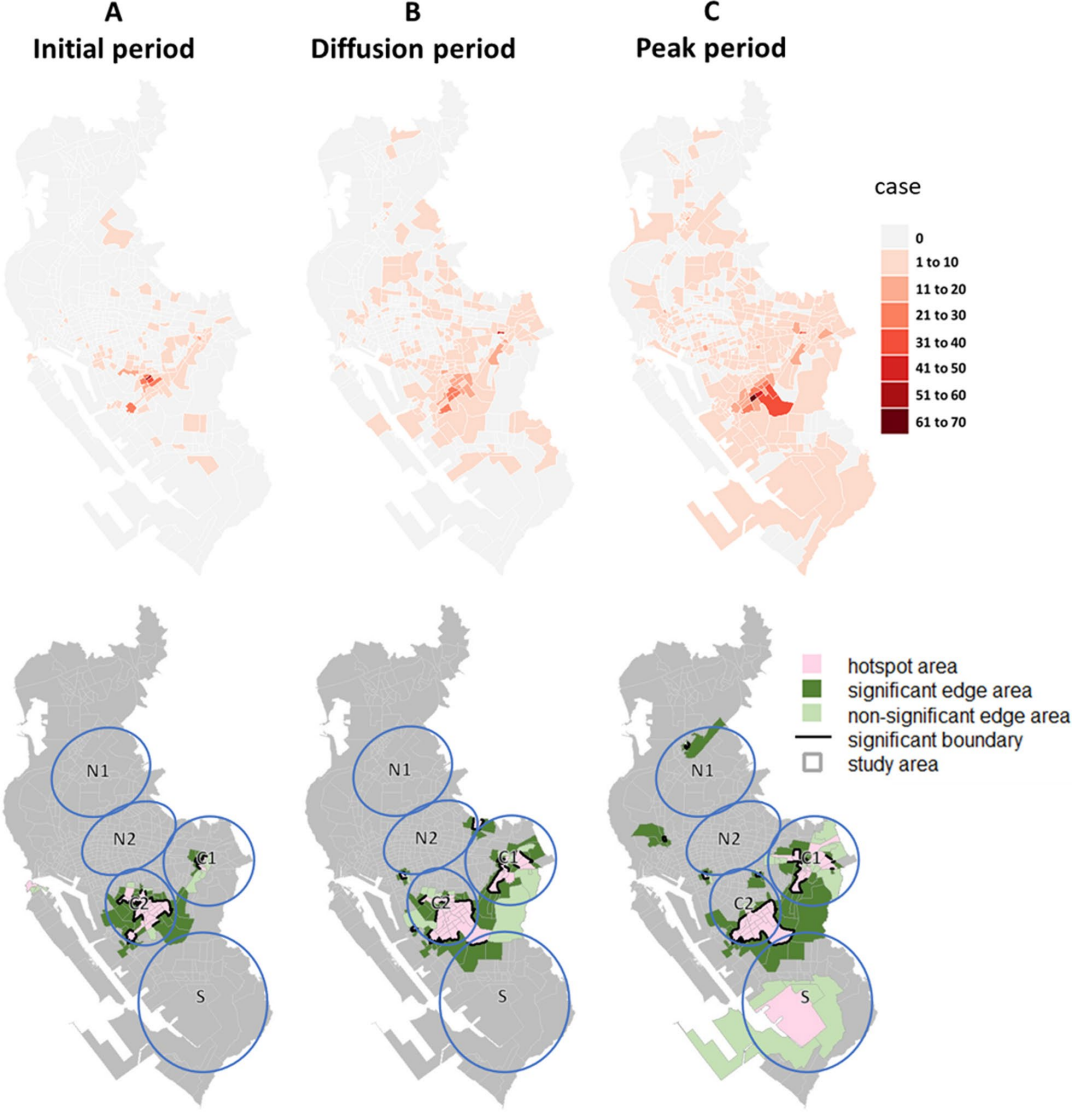
Spatial distribution of dengue cases and delimited areas: 2002

**Fig. 9:**
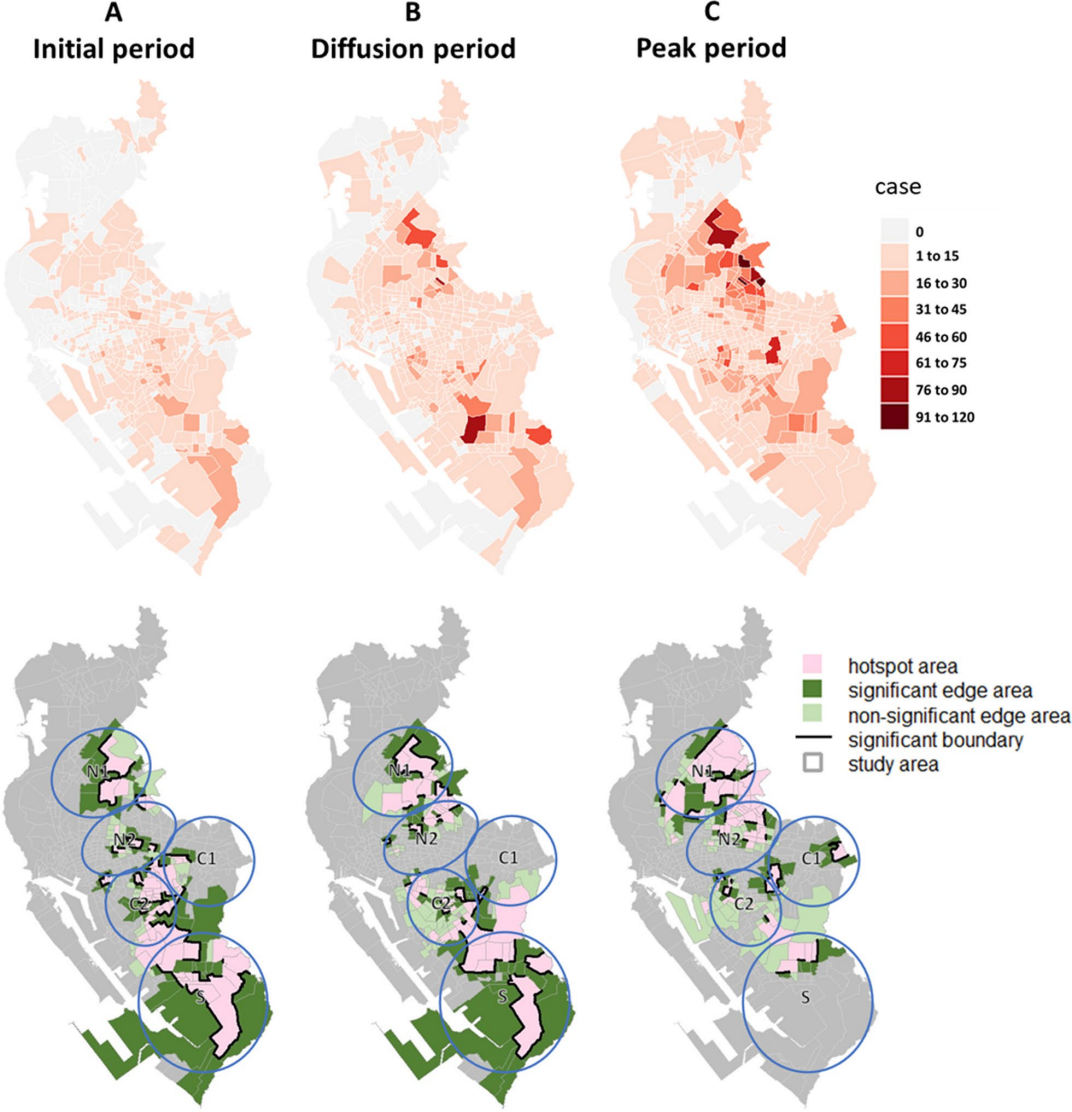
Spatial distribution of dengue cases and delimited areas: 2014

**Fig. 10:**
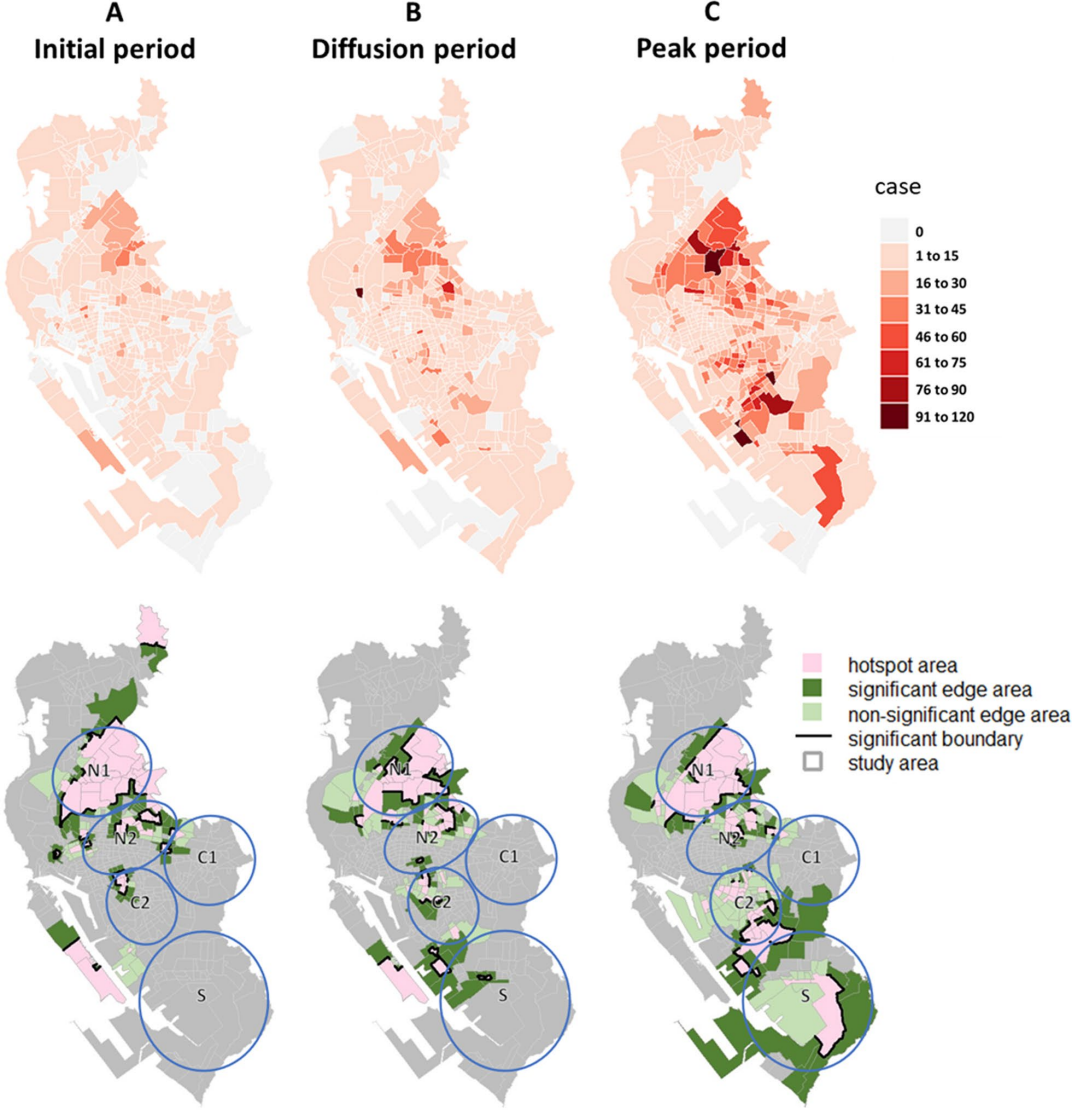
Spatial distribution of dengue cases and delimited areas: 2015

Regarding the diffusion patterns of dengue epidemics, in 2002, hotspots appeared in Regions C1 and C2 in all three periods and grew larger, forming significant hotspot boundaries over time. A hotspot also appeared in the peak period in Region S, as cases were concentrated there (Fig. 8). In 2014 and 2015, the hotspot areas were larger compared to 2002. In 2014, the cases spread in Regions N1 and N2, resulting in broader hotspot areas and more significant hotspot boundaries. Additionally, hotspots and significant hotspot boundaries were detected at the junction of Regions C1 and C2. The southern Region S continued to spread during the initial and diffusion periods but became less severe in the peak period (Fig. 9). In 2015, the spread of the epidemic showed a noticeable trend from the north to the south of the study area, with cases spreading in Regions N1, N2, and C2 and significant hotspot boundaries detected. Region S also became a hotspot area with significant hotspot boundaries in the peak period (Fig. 10).

Table 1 displays the incidence rates and number of dengue cases in three distinct areas during severe dengue outbreaks. The hotspot areas had the highest average incidence rates during all epidemic stages. Meanwhile, the incidence rates in the significant edge areas were lower during the pre-stage period. This shows a marked difference in risk between the hotspot and significant edge areas and that significant boundaries likely existed along the borders of these two areas. The incidence rates in the edge areas increased during both the exponentially-rising and continuously-rising stages, but the incidence rate trends in hotspot areas flattened out.

**Table 1:** Descriptive statistics for each differentiated area

Serious year	2002			2014			2015		
	Hotspot area	Sig. edge area	Non-sig. edge area	Hotspot area	Sig. edge area	Non-sig. edge area	Hotspot area	Sig. edge area	Non-sig. edge area
*Exponentially-rising stage*									
Incidence rate: period A	391.84 (92.61)	12.80 (4.00)	43.51 (17.37)	305.63 (26.49)	54.24 (7.10)	92.06 (14.96)	422.98 (64.89)	108.81 (14.51)	151.84 (30.25)
Incidence rate: period B	439.27 (144.41)	65.82 (14.49)	127.36 (35.99)	268.84 (39.05)	185.73 (23.03)	278.81 (46.23)	400.17 (47.30)	343.79 (44.78)	336.02 (55.31)
N	27	33	9	63	76	33	50	75	35
*Continuously-rising stage*									
Incidence rate: period B	382.31 (98.17)	27.47 (6.37)	61.97 (13.38)	553.88 (61.45)	123.96 (12.81)	260.76 (37.26)	640.88 (80.93)	244.25 (31.57)	262.87 (23.15)
Incidence rate: period C	393.74 (91.75)	64.30 (9.43)	63.83 (15.86)	594.92 (77.40)	298.03 (37.70)	474.85 (61.07)	629.70 (51.61)	575.44 (58.24)	723.65 (56.25)
N	40	44	12	56	68	50	46	57	47
*Declining stage*									
Incidence rate: period C	521.47 (104.39)	63.74 (8.87)	99.31 (20.81)	822.64 (76.25)	222.61 (20.98)	430.42 (37.25)	881.98 (58.70)	380.35 (31.43)	582.19 (37.93)
Incidence rate: period D	291.91 (90.08)	108.98 (21.66)	94.27 (22.14)	303.93 (30.84)	203.43 (24.97)	212.88 (20.88)	315.35 (21.31)	233.54 (22.85)	296.02 (26.93)
N	35	49	22	58	54	61	63	61	77

Note 1: Standard errors in parentheses

Note 2: N is the number of villages in the specific area

### Comparing incidence rates using DID estimators

The incidence rate is used to track changes in transmission risk over time. Tables 2, 3 and 4 present the results of the DID regression models for three comparison groups in different years. The ρ in the tables represents the spatial autoregressive parameters that control the existing spatial autocorrelation if the coefficient is significant.

**Table 2:** DID regression models between significant edge areas and hotspot areas

	Exponentially-rising stage			Continuously-rising stage			Declining stage		
	2002	2014	2015	2002	2014	2015	2002	2014	2015
Intercept	420.42*** (67.20)	281.14*** (23.65)	432.13*** (44.95)	403.73*** (76.39)	553.94*** (57.03)	653.32*** (51.21)	414.43*** (72.40)	562.30*** (46.36)	600.92*** (42.37)
*SIG.EDGE*	− 403.60*** (98.25)	− 228.70*** (30.25)	− 339.18*** (53.44)	− 383.75*** (90.38)	− 403.21*** (59.53)	− 438.97*** (67.67)	− 365.33*** (84.57)	− 386.66*** (54.86)	− 354.83*** (48.31)
TIME* *SIG.EDGE* (DID estimator)	44.71 (90.03)	138.52*** (34.01)	262.55*** (49.36)	45.93 (35.47)	215.12*** (51.32)	385.08*** (72.08)	69.78 (40.43)	133.52^ (68.76)	146.36* (62.08)
N	60	139	125	84	124	103	84	112	124
ρ	0.015 (0.0296)	0.072*** (0.0165)	0.070*** (0.0195)	0.056* (0.0230)	0.098*** (0.0154)	0.068** (0.0231)	0.055* (0.0249)	0.123*** (0.0145)	0.138*** (0.0141)

**Table 3:** DID regression models between non-significant edge areas and hotspot areas

	Exponentially-rising stage			Continuously-rising stage			Declining stage		
	2002	2014	2015	2002	2014	2015	2002	2014	2015
Intercept	430.50*** (93.10)	288.13*** (26.73)	456.90*** (51.05)	435.31*** (112.48)	553.98*** (63.77)	643.76*** (43.50)	428.66*** (96.42)	571.27*** (46.53)	612.90*** (45.72)
*NON-SIG.EDGE*	− 366.77^ (202.70)	− 180.48*** (48.78)	− 260.27*** (73.01)	− 284.63 (178.21)	− 294.42*** (81.44)	− 371.38*** (70.47)	− 285.89* (128.47)	− 195.56** (59.86)	− 135.13** (50.79)
TIME* *NON-SIG.EDGE* (DID estimator)	64.36 (221.46)	156.37** (58.53)	163.32* (71.20)	− 7.80 (79.78)	248.40*** (70.10)	442.54*** (76.44)	34.65 (65.46)	− 56.52 (73.34)	− 56.74 (59.04)
N	36	96	85	52	106	93	57	119	140
ρ	0.037 (0.0495)	0.067** (0.0245)	0.106*** (0.0293)	0.098** (0.0339)	0.100*** (0.0200)	0.0217 (0.0357)	0.088** (0.0340)	0.123*** (0.0150)	0.132*** (0.0139)

**Table 4:** DID regression models between significant and non-significant edge areas

	Exponentially-rising stage			Continuously-rising stage			Declining stage		
	2002	2014	2015	2002	2014	2015	2002	2014	2015
Intercept	77.28*** (17.87)	174.05*** (25.19)	248.87*** (38.00)	65.23*** (12.35)	355.72*** (40.29)	487.44*** (44.82)	97.51*** (20.23)	314.25*** (25.21)	439.97*** (31.64)
*SIG.EDGE*	− 61.12** (22.32)	− 102.79*** (30.66)	− 128.58* (49.97)	− 38.35** (13.80)	− 200.68*** (53.78)	− 202.75** (64.12)	− 35.94 (25.50)	− 102.18** (38.41)	− 95.03* (46.06)
TIME* *SIG.EDGE* (DID estimator)	39.70^ (20.47)	96.40** (29.66)	219.22*** (51.13)	35.01*** (10.53)	157.85*** (47.66)	274.76*** (72.68)	49.88* (20.52)	3.92 (41.03)	− 41.04 (48.86)
N	42	109	110	56	118	104	71	115	138
ρ	0.161*** (0.0481)	0.144*** (0.0279)	0.093** (0.0318)	0.090* (0.0431)	0.127*** (0.0263)	0.120*** (0.0267)	0.067^ (0.0340)	0.104*** (0.0273)	0.139*** (0.0236)

Table 2 compares the incidence rate in hotspot areas (GROUP = 0) with significant edge areas (GROUP = 1) in severe years. The SIG.EDGE coefficients are significantly negative, suggesting a more severe outbreak in hotspot areas compared to significant edge areas. The interaction between TIME and SIG.EDGE, as represented by the DID estimators TIME*SIG.EDGE, reveals that the growth rate of significant edge areas is significantly higher than hotspot areas during all three stages in 2014 and 2015.

Table 3 compares the incidence rate in hotspot areas (GROUP = 0) with non-significant edge areas (GROUP = 1). The NON-SIG.EDGE coefficients are significantly negative at all stages in the three years, showing that hotspot areas have a higher risk overall compared to non-significant areas. The DID estimators are significantly positive during the exponentially-rising and continuously-rising stages in 2014 and 2015, indicating an increase in growth rate in non-significant edge areas.

Table 4 compares the incidence rate in non-significant edge areas (GROUP = 0) with significant edge areas (GROUP = 1). Unlike the previous two comparisons, this one assesses the significant difference in the level of increase or decrease between significant edge areas and non-significant edge areas. The SIG.EDGE coefficients are all significantly positive, indicating a higher incidence rate in significant edge areas than in non-significant edge areas across all stages. Except for the declining stages in 2014 and 2015, all DID estimators are significantly positive during exponentially-rising and continuously-rising stages, indicating a higher growth of incidence rate in significant edge areas compared to non-significant edge areas.

In summary, significant edge areas have a higher and more significant upward trend compared to hotspot areas on average throughout all time, particularly during the rising stages of 2014 and 2015. Non-significant edge areas exhibit the same tendency. The change in risk is more remarkable in significant edge areas than in non-significant edge areas. These findings support the hypothesis that regions on the edge of hotspots experience a larger increase in risk compared to the hotspot itself, particularly in significant edge areas. Furthermore, during declining stages, the risk in significant edge areas decreases faster than in hotspot areas, indicating a higher degree of recovery in these areas.

### Results of sensitivity analysis

**Fig. 11:**
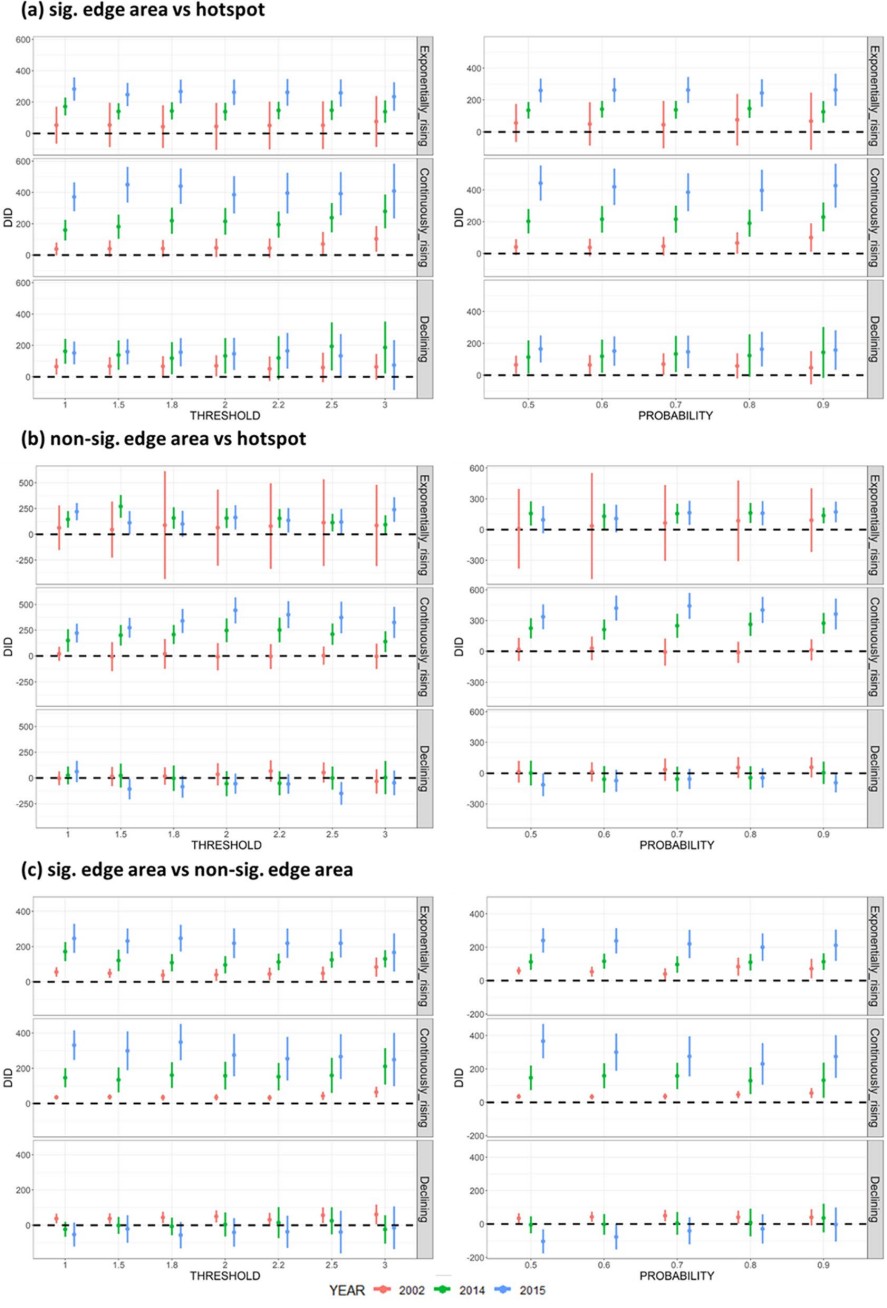
Sensitivity analysis of 90% CI of the DID estimators

Figure 11 depicts DID estimators' 90% confidence intervals (CIs) at different parameter settings in the three selected severe years. The left side of the figures indicates the values for the boundary and hotspot detection thresholds (c), ranging from 1.0 to 3.0, and the probabilities are fixed at 0.7. Notably, at c = 1.0, approximately 19% of villages were identified as hotspots, whereas at c = 3.0, only an average of 5% of villages were classified as hotspot areas. For reference, approximately 9% of villages were captured as hotspots when c = 2.0. On the other hand, the thresholds are fixed to be 2.0 in the right side of the figures, but the probabilities to determine the hotspots and boundaries are set between 0.5 and 0.9.

Figure 11a is the DID estimators comparing the significant edge area and the hotspot, and the results show the estimators are consistent in different settings of threshold and probability. All DID estimators are positively significant in 2014 and 2015 during the exponentially-rising and continuously-rising stages; 90% CI of the DID estimators in 2002 during all stages; and the other two years during the declining stage are on the edges of significance. This suggests that the significance remains consistent in the aforementioned stages when the thresholds range from 1 to 3 times the average number of dengue cases in the pre-periods. Figure 11b is the DID estimators comparing between the non-significant edge area and the hotspot, and the results from different settings of thresholds and probabilities are consistent. The result implies that all DID estimators are positively significant during the exponentially-rising and continuously-rising stages in 2014 and 2015, but not significant during all stages in 2002 and the declining stage in all selected years. Last, Fig. 11c shows the comparisons between the two types of edge areas, and the results of the two kinds of parameter settings remain consistent. The DID estimators are all significantly positive in all selected years during the exponentially-rising and continuously-rising stages but not significant in the declining stages, with coefficients in 2002 on the edges of significance.

The overall results show that regardless of how the threshold or probability value is set, the value of the DID may change. Still, the overall significance of the DID remains unchanged. Also, when fixing the threshold or probability and changing one of their parameters, the distributions of 90% CI are similar, which means that the results of DID estimators are also consistent.

## Discussion

This study delimits the hotspot and its edge areas and examines the risk at the edges of disease hotspots during outbreaks. Our results indicate that the growth rate of risk in the edge areas would become higher than in the hotspot areas. During the exponentially-rising and continuously-rising stages, the risk change in both significant and non-significant edge areas increased more than in the hotspot areas in 2014 and 2015. Moreover, there are substantial differences between the two types of edge areas. The risk increase in significant edge areas is larger than in non-significant edge areas. In general, the incidence rate trends are increasing in the edge areas, while the trends are flattened out or even decreasing in the hotspot. The higher risk increase in the edge areas could be explained by the higher intensity contact frequency between susceptible and infected people in the surrounding regions of hotspots. The findings support the hypothesis that areas outside of hotspots would have a more significant increase in disease risk than hotspots, leading to a higher risk of disease transmission and potential disease foci.

### Mechanism of disease diffusion

The growth rate of the incidence rate in edge areas is significantly larger than in hotspot areas during the exponentially-rising and continuously-rising stages (Tables 2, 3), showing a process of expansion diffusion. This suggests that based on the human movement taken into account by the adjacency in the CAR model, the edge areas may grow more serious and lead to the spread of the disease. When people move across villages, infections can be brought to the edge areas separately, creating new disease foci and making edge areas more dangerous due to the larger proportion of susceptible individuals present.

The spread of a vector-borne disease could occur from a single focus. However, when human mobility is factored in, the disease can propagate from multiple foci [3], and the spread often occurs within a 1 km radius of clusters [32]. That is, these new foci would emerge in the edge areas, following the expansion type of disease diffusion, wherein the periphery would become more serious overtime [12]. The same findings hold when examining the situation from an individual perspective, particularly superspreaders. These individuals are the drivers of disease transmission, moving from the core of hotspots to edge areas during the exponential growing stage [26]. Moreover, asymptomatic infected individuals, often highly mobile adults, can spread the disease easily during movement [24], potentially sparking future outbreaks [8]. This further underscores the risk of disease transmission at the edges of hotspots.

In contrast, hotspot areas exhibit either stagnation or a decrease in their incidence rates across all stages, as shown in Table 1. Notably, the reduced risk in hotspot areas from the peak (C) to under-control (D) periods aligns with the overall declining trend in the study area. This is due to the strategic allocation of disease control measures, focusing primarily on hotspot regions acroding to the dengue control guidelines (Taiwan CDC, 2019).

While the entire study area experiences increasing trends during the exponentially-rising and continuously-rising stages, risk within hotspot areas either remains stable or decreases. This pattern follows the concept of relocation diffusion, indicating that the original disease hotspots are weakening while edge areas become more vulnerable [12]. These findings suggest that hotspots may be approaching a point of saturation during peak periods, emphasizing the need to shift more attention and resources toward the edges of hotspots.

In summary, the rise of incidence rate in edge areas reflects a form of short-distance expansion diffusion. In contrast, the flat or decrease in incidence rates in the hotspot areas indicates the weakening of the original hotspots referred to by the short-distance relocation diffusion. Therefore, the spread of dengue in this study shows a mixed diffusion type, a common type of disease diffusion including both expansion and relocation [12]. The importance of edge areas lies in the fact that the center of diffusion shifts over time while the hotspot expands around without the same speed. This would lead to non-concentric movement of the hotspot and short-distance relocation diffusion, enabling the disease to spread outward. Hence, the current edge area could transition into the new diffusion source at a subsequent time, highlighting the need for preventive measures at the edges of hotspots to manage the associated risks.

### Human mobility

This study assumes that people moving within a short distance around nearby villages can reflect the major human mobility of disease spreads. Based on this assumption, human mobility is defined as Rook contiguity—neighbors are two villages sharing the same border—to be the adjacent matrix representing the spatial structure in the CAR model. Also, the proximity of this study is confined to the vicinity of geographic space in terms of the edge area. It is assumed that people only move between neighborhoods over short distances in dense urban areas, implying that infections could occur at home or through daily activities. The dominant species in the study area is Aedes aegypti, which prefers indoor and urban environments [13, 14]. This causes the spread to occur within short distances. The spread of dengue on a fine scale mainly concentrates in a household range smaller than 50 m [29], while another study shows that 97% of transmission occurs within 1 km when the average distance between households is 382 m [11]. Also, the mean distance between each case in the same study area is around 500 m [16], compared to this study that showed the average centroid distance between a village and its neighbors is 592.7 m, indicating short distance adjacency, Rook contiguity represents similar fine-scale proximity to the above studies.

### Policy implications

Based on our findings, the significance of the the edges of hotspots becomes apparent mainly in the context of exceptionally large-scale outbreak years. Consequently, it is imperative to emphasize the importance of incorporating edge areas into epidemic prevention policies. Accroding to the current prevention and control policies, which predominantly center on communities with epidemic clusters [30, 34], the edge areas may inadvertently be overlooked in epidemic prevention efforts. While the targeting of hotspots, typically characterized by the highest incidence rates, remains indispensable, an expanded focus on edge areas, especially significant edge areas, is equally imperative. Thus, it is recommended that health authority prioritize the allocation of resources and attention to these edge areas within the context of dengue control. This emphasis gains heightened significance owing to the unique diffusion patterns found in edge areas, patterns that frequently correlate with an increased risk of disease transmission. Such a strategic approach becomes especially pivotal when developing fine-scale disease control strategies to ensure a more effective response to extremely dengue outbreaks.

### Limitations

There are several limitations to this study. First, several time-varying factors that may influence the incidence rate in a period, such as climate factors and vector indicators, were not considered when computing DID estimation. Second, neighbors in only the study area are defined in the adjacency matrix of the CAR models since this study only focuses on the urban area. Low density along the outside edges of the study area which implies less movement between the study area to its edges. However, there still could be an edge effect, and long-distance movement could also affect the spread. It warrants further investigation for considering movement-based proximity to reflect actual human mobility, such as the population flow based on the daily commute. Last, epidemics are cut into periods manually in this study. Advanced time series statistical analysis could capture the characteristics of the epidemic over time in more detail.

## Conclusion

This study statistically examines the risk increase at the edges of disease hotspots during large-scale outbreaks. Our results have confirmed that in years characterized by exceptionally large-scale outbreaks, the edge areas of a hotspot exhibit a heightened risk of disease transmission when the disease is spreading rapidly. New disease foci could appear in the edge areas via transmission while the risk in the hotspot gradually decreases. This finding explains the geographic diffusion mechanism of epidemics, a pattern mixed with expansion and relocation, indicating that the edge areas play an essential role. Most current policies only focus on the hotspot itself, whereas the edges of the hotspot are potential blind spots for disease control. Therefore, spatial targeting of innervations may pay more attention to the edges of disease hotspots to prevent the subsequent epidemic spread.

## Data Availability

The data is available on request through the corresponding author.
